# Editorial: Biomedical Data Visualization: Methods and Applications

**DOI:** 10.3389/fgene.2022.890775

**Published:** 2022-04-27

**Authors:** Tianzhi Wu, Chuan-Le Xiao, Tommy Tsan-Yuk Lam, Guangchuang Yu

**Affiliations:** ^1^ Department of Bioinformatics, School of Basic Medical Sciences, Southern Medical University, Guangzhou, China; ^2^ State Key Laboratory of Ophthalmology, Zhongshan Ophthalmic Center, Sun Yat-sen University, Guangzhou, China; ^3^ State Key Laboratory of Emerging Infectious Diseases, School of Public Health, The University of Hong Kong, Pokfulam, Hong Kong SAR, China; ^4^ Laboratory of Data Discovery for Health Limited, Hong Kong, Hong Kong SAR, China

**Keywords:** biomedical data, visualization, transcriptomics, virtual reality, network visualiztion

“A picture tells a thousand words.” This is very true in many circumstances but particularly for modern academia as a nicely illustrative figure grabs our attention and helps us explain scientific findings. Data visualization is the most effective way to explain and convey rich information, especially for complex biomedical data. The rapid growth of biomedical data in both volume and complexity creates new challenges in presenting data effectively and accurately (O’Donoghue et al., 2018). This includes exploring data to reveal hidden information and presenting analysis findings. New visualization methods are being developed to address new emerging problems or provide new insights into old data types. Innovations in visualization will continue to revolutionize how we learn from our data, and it is extremely important for biomedical research.

Scatter plots and line charts are the most basic form of data visualization through simply mapping variables to data points. Bar charts, histograms, boxplots, and heatmaps are also widely used. These methods and their combination solve most of the visualization needs, including presenting data overview or summary and helping us to identify patterns or trends. With the rapid growth data volume, effectively utilizing plotting space becomes much in demand. For example, how to truncate an overly long chart, rearrange and display the key parts of a graph in the limited space for publication, and how to select parts of them to zoom in or zoom out while keeping the panorama of the current chart. These are the details that need to be resolved. The ggbreak package can be used as an example in this respect (Xu et al.). It was designed to solve the above issues, by increasing the available visual space for a better presentation of the data and detailed annotations and thus improves our ability to interpret the data. The ggbreak package is consistent with the ggplot2 package by following the syntax of the grammar of graphics (Wilkinson, 2010) and implementing such syntax. There is no additional learning cost to use ggbreak if users are familiar with the ggplot2 syntax. Another package we introduce here is smplot (Min and Zhou), which is also based on R and ggplot2, it simplifies the plotting process of commonly statistical graphs for easy visualize, such as violin plot, slope chart, raincloud plot, and so on. These tools reflect a trend in the development of basic tools: solving practical problems while no additional cost is added and keeping it a good user experience.

It is always right to choose the appropriate visualization method according to specific needs. The Venn diagram can efficiently reflect the relationship between multiple sets. Therefore, it is often used to distinguish members of gene sets, pathways, species, etc. When the number of sets is less than 5, the Venn diagram is a more intuitive form of data visualization than heatmap or tables. This is also the reason why the Venn diagram appears frequently in biomedical research publications. The ggVennDiagram package (Gao et al.) has been developed as a systematic and easy-to-use method for calculating overlapping members in different sets and visualizing such intersection information in Venn diagrams based on the ggplot2 syntax. It has some features that are not available in general tools, including novel shapes and color filling of different proportion regions. When we can provide new features and perspectives, it is sometimes necessary to reinvent the wheel. The tools have been continuously improved during such overthrows.

When discussing biomedical visualization, it is more than a direct display of given data. Usually, the data sets in the modern biomedical field are complex. Before coming to results and conclusions, scientists spend most of their time processing and exploring data. Therefore, many tailored visualization tools have been developed to meet the needs of data exploration. For example, there is a growing demand in different biomedical research scenarios for network visualization of the relationship between different types of nodes with complex metadata. Integrating different attribute information of nodes and edges in a network may inspire new insights. The CrossLink package (Liu et al.) was designed to plot a network diagram with node attributes as graph annotation aligned to the network. The HandyCNV package (Zhou et al.) is also developed for a specific need. It provides common functions for CNV (copy number variant) and ROH (runs of homozygosity) research, including basic data processing and also essential visualization. Designed as a one-stop tool, HandyCNV aims to make analysis easier and more efficient. Similarly, in the field of microbiome, an easy-to-use tool called EasyMicroPlot (Liu et al.) provides analysis and visualization for clinical microbial studies. Overall, these tools reflect the current needs of visualization methods: to develop standardized, time-saving, and user-friendly one-stop tools for corresponding scenarios.

Data from clinical registrations can provide more insights into patients’ treatments and their outcomes. This is what we called real-world data, which goes beyond the controlled clinical trial, and allows us to test the results in an uncontrolled reality world (Rudrapatna and Butte, 2020). However, to produce credible conclusions, there are still many aspects that need to be improved. The most basic problem is the missing data. It is hard to guarantee the completeness of real-world data collection, and the incomplete data will pose challenges for further data analysis tasks. Therefore, it is important to handle these missing data in an appropriate way. ImputEHR (Zhou and Saghapour) evaluates the influence of various imputation approaches in real-world data sets such as EHR (Electronic Health Record) and provides a practical and fast imputation tool. This tool provides a web application, which makes the operation and visualization interactive. Interactivity is a new trend in the development of visualization tools. It can significantly improve the user experience of exploring data.

Among the emerging technologies in recent years, virtual reality (VR) is more and more widely used in medical fields, typical include in medical education and training. The advantage is that VR can dynamically explore complex biomedical data. But on the other hand, it is also limited by expensive hardware and complex data preprocessing steps. SinglecellVR is a web application that utilizes VR to visually explore single-cell data and common sequencing data, including transcriptome, epigenome, and proteome data (Stein et al.). It is designed for cheap and easily available virtual reality hardware, such as Google Cardboard. As a new solution of single-cell visualization, SinglecellVR has been reported within several media, which reflects the interest and concern of researchers on the use of low-cost VR in biomedicine. With the increase of data dimensions, VR will have a wide range of applications in exploring biomedical data.

Spatial transcriptomic is a popular molecular measurement technology used in the biomedical field recently. It can measure transcriptional information while retaining tissue spatial information. A review on the analysis and visualization of spatial transcriptomic data is presented in this research topic (Liu et al.). It covers the latest status of spatial transcriptome technology, and mainly focus on the current analysis and visualization tools in the preprocessing of data, the identification of spatially related gene patterns, and the visualization in expression domain, spatial domain, and cell-to-cell communication. As the latest research field of biomedicine, spatial transcriptomic is expected to reveal the complex transcriptional structure of heterogeneous tissues and enhance our understanding of the cellular mechanism of the disease (Burgess, 2019). Thus, more and more visualization methods and tools suitable for this field should be developed.

We have screened parts of the current biomedical visualization tools and their focus points cover different scenarios, from basic visual representation to specific field applications. They could be presented as part of the status of biomedical data visualization and their design concept also reflect the current trend of visualization. Moreover, biomedical data visualization methods play important roles in exploring and visualizing large-scale omics data, including genomics, transcriptomics, epigenetics, quantitative imaging, and so on. Regardless of the changes in data generation techniques, visualization tools, application scenarios, and research areas, the main tasks and development trends of data visualization remain unchanged—to better explore data, to better represent results, to improve the efficiency of information sharing and communication, and to improve user experience.
